# Automated C. elegans behavior analysis via deep learning-based detection and tracking

**DOI:** 10.1371/journal.pcbi.1013707

**Published:** 2025-11-11

**Authors:** Xiaoke Liu, Jianming Liu, Wenjie Teng, Yuzhong Peng, Boao Li, Xiaoqing Han, Jing Huo

**Affiliations:** 1 School of Basic Medical Sciences, Shandong Second Medical University, Weifang, Shandong, China; 2 Weifang Key Laboratory of Collaborative Innovation of Intelligent Diagnosis and Treatment and Molecular Diseases, School of Basic Medical Sciences, Shandong Second Medical University, Weifang, Shandong, China; 3 School of Life Science and Technology, Shandong Second Medical University, Weifang, Shandong, China; Xinjiang Technical Institute of Physics and Chemistry, CHINA

## Abstract

As a well-established and extensively utilized model organism, Caenorhabditis elegans (C. elegans) serves as a crucial platform for investigating behavioral regulation mechanisms and their biological significance. However, manually tracking the locomotor behavior of large numbers of C. elegans is both cumbersome and inefficient. To address the above challenges, we innovatively propose an automated approach for analyzing C. elegans behavior through deep learning-based detection and tracking. Building upon existing research, we developed an enhanced worm detection framework that integrates YOLOv8 with ByteTrack, enabling real-time, precise tracking of multiple worms. Based on the tracking results, we further established an automated high-throughput method for quantitative analysis of multiple movement parameters, including locomotion velocity, body bending angle, and roll frequency, thereby laying a robust foundation for high-precision, automated analysis of complex worm behaviors. including movement speed, body bending angle, and roll frequency, thereby laying a robust foundation for high-precision, automated analysis of complex worm behaviors. Comparative evaluations demonstrate that the proposed enhanced C. elegans detection framework outperforms existing methods, achieving a precision of 99.5%, recall of 98.7%, and mAP50 of 99.6%, with a processing speed of 153 frames per second (FPS). The established framework for worm detection, tracking, and automated behavioral analysis developed in this study delivers superior detection and tracking accuracy while enhancing tracking continuity and robustness. Unlike traditional labor-intensive measurement approaches, our framework supports simultaneous tracking of multiple worms while maintaining automated extraction of various behavioral parameters with high precision. Furthermore, our approach advances the standardization of C. elegans behavioral parameter analysis, which can analyze the behavioral data of multiple worms at the same time, significantly improving the experimental throughput and providing an efficient tool for drug screening, gene function research and other fields.

## Author summary

Studying the behavior of the worm C. elegans is crucial for understanding genetics and neurobiology, but manually tracking its movements is slow and impractical for large experiments. To overcome this, we developed an automated, high-speed system that uses advanced deep learning to accurately detect and track worms in real-time, even when they temporarily hide or touch each other. Our approach enhances the YOLOv8 architecture by incorporating a Convolutional Block Attention Module, which enables the model to focus on the most relevant visual features of the worms while suppressing background interference. Additionally, we modified the loss function to better handle the detection of small and overlapping worms, significantly improving localization accuracy. These technical innovations allow our method to follow multiple worms and automatically measure key behaviors like movement speed, body bending, and roll frequency with exceptional precision. By providing a robust and standardized algorithm, making advanced behavioral studies accessible to more biologists.

## 1 Introduction

The nematode C. elegans, characterized by their transparent bodies, offer an exceptional experimental model for biological research. This unique transparency facilitates direct observation of internal structures and cellular dynamics, enabling real-time microscopic examination of fundamental processes such as cell division and organismal development. As a premier model organism, worms combine several advantageous features: straightforward maintenance and handling protocols, coupled with versatile genetic manipulation capabilities through both forward and reverse genetics approaches [1]. These distinctive characteristics collectively establish worms as an invaluable system for diverse scientific investigations, particularly in developmental biology and cellular research.

Behavioral analysis serves as a fundamental research paradigm in multiple scientific disciplines, particularly in neuroscience and genetics. The behavioral study of C. elegans worms has emerged as a significant research focus in contemporary biology. Systematic monitoring and quantification of C. elegans locomotor behavior have enabled substantial advancements in several research domains, including pharmacological screening [2], ecotoxicological assessment [3], anti-aging investigations [4], and human disease modeling [5]. These behavioral-based approaches have provided critical scientific insights and established robust experimental frameworks for understanding human health maintenance and longevity mechanisms [6–8].

The accurate identification and differentiation of individual worm trajectories are essential prerequisites for all aforementioned studies. Furthermore, the analysis of worm behavioral performance necessitates precise quantification of movement parameters, including movement speed, bending angle, and roll frequency. Consequently, the development of reliable worm detection and tracking methodologies has emerged as a critical component in behavioral research. As a predominant model organism in biological research, C. elegans has facilitated the extensive application of digital image processing techniques in various experimental studies. These applications encompass microscopic image segmentation, phenotypic quantification, three-dimensional atlas reconstruction, and automated behavioral tracking, significantly advancing worm-related research methodologies [9].

In early studies, investigators primarily utilized traditional image processing approaches for worm tracking studies. Swierczek et al. (2011) designed a real-time computer vision system, the Multi-Worm Tracker (MWT), capable of simultaneous quantitative behavioral analysis of multiple C. elegans worms individuals at video rate resolution [10]. Husson et al. (2013) developed a video processing tool for nematode tracking that quantifies motion parameters but requires extensive manual annotation [11]. Wang et al. (2013) introduced Track-A-Worm, a functionally rich, open-source system characterized by user-friendly operation [12]. Itskovits et al. (2017) presented a Multi-Animal Tracker (MAT), offering an end-to-end, user-friendly solution for simultaneous imaging, tracking, and analysis of complex multi-animal behaviors. The system’s core incorporates a machine learning algorithm that enables flexible imaging across diverse experimental conditions and model organisms [13]. Javer et al. (2018) established a comprehensive platform for C. elegans behavioral data, featuring a large public database on Zenodo, a versatile data interchange format (WCON), and open-source software for analysis [14]. This invaluable resource underscores the importance of reproducible workflows. Most notably, Javer et al. (2018) make a valuable contribution by developing a new set of interpretable, handcrafted features to compactly quantify C. elegans behavior and to bridge the gap between the high performance of machine learning and the need for biological interpretability [15].

However, traditional image processing algorithms exhibit substantial performance degradation under increasingly sophisticated experimental setups. Furthermore, as experimental datasets continue to expand exponentially [16], manual counting and labeling become progressively more challenging. Consequently, high-throughput automated methodologies have emerged as the predominant approach, effectively replacing conventional image processing techniques in contemporary research.

Deep learning methodologies leverage the computational power of convolutional neural networks to autonomously extract object features while preserving spatial relationships, making these models particularly suitable for behavioral research applications. The integration of deep learning with computer vision techniques enables more efficient and precise processing and analysis of biological images [17,18], offering promising solutions to the aforementioned challenges. Andre et al. (2022) introduced NemaNet, conducting a comprehensive comparative analysis with thirteen established CNN architectures, while also releasing NemaDataset - a public repository containing 3,063 annotated microscopic images spanning five worm species [19]. Walter et al. (2021) established an efficient, user-friendly tracking system capable of simultaneous monitoring of multiple individuals, achieving real-time (60 Hz) tracking performance for populations of up to 256 organisms through advanced background-subtraction techniques [20]. Pereira et al. (2019) created LEAP (LEAP Estimates Animal Pose), an innovative deep learning framework for animal pose estimation, which integrates a graphical interface for body part annotation and network training [21]. Notably, Bates et al. (2022) successfully employed a Faster R-CNN framework to achieve robust identification and detection of C. elegans across various life stages in complex environments, showcasing its advantages in speed, accuracy, and generalizability [22]. Banerjee et al. (2023) designed Deep-Worm-Tracker, an end-to-end deep learning model demonstrating high accuracy and real-time inference capabilities. Thevenoux et al. implemented a hybrid approach combining custom computer vision algorithms with convolutional neural networks for species identification in quarantine worm imaging [23]. Weheliye et al. (2024) recently developed Deep Tangle Crawl, a deep learning model specifically designed to resolve worm identities and maintain continuous trajectories during collisions and coils, thereby enabling the study of social behaviors and complex morphologies [24].

The aforementioned research primarily employs object detection algorithms to initially identify worms and subsequently track their movements. Object detection, a fundamental task in computer vision, focuses on identifying all objects of interest within an image or video while determining their respective categories and spatial coordinates. Recent advancements have led to significant progress in end-to-end object detection algorithms, which unify feature extraction, region proposal, and classification-regression processes within a single neural network architecture, enabling seamless training and inference. Notable examples of these advanced algorithms include the YOLO series (YOLOv1 [25] through YOLOv8 [26]) and SSD [27] architectures. Similarly, object tracking represents a fundamental task in computer vision, focusing on the localization and continuous monitoring of specific objects within video sequences. Before the advent of deep learning, conventional object tracking methodologies predominantly relied on manually engineered features, typically employing Kalman filters or particle filters for state estimation. However, these approaches exhibited limited generalization capabilities. The emergence of deep learning techniques has driven substantial advancements in the field of object tracking. Banerjee et al. (2023) implemented an integrated approach combining YOLOv5 [28] with StrongSORT for real-time multi-worm detection and tracking [23]. However, the system demonstrates significant limitations in tracking performance during target occlusion or disappearance events. Furthermore, StrongSORT’s dependency on high-quality detection outputs and computationally intensive appearance feature extraction and matching processes results in substantial computational overhead, consequently affecting real-time processing efficiency.

Addressing the aforementioned challenges, we have developed a modified worm detection and tracking framework that integrates YOLOv8 with ByteTrack [29]. YOLOv8 achieves superior detection accuracy through architectural optimizations, refined training strategies, and advanced data augmentation techniques, demonstrating exceptional performance in small target detection tasks such as worm identification. The architecture maintains high precision while optimizing inference speed, rendering it particularly suitable for real-time applications. To further improve detection accuracy, we have enhanced YOLOv8 by incorporating an attention mechanism module [30,31] and optimizing the loss function. ByteTrack implements a dual-stage matching strategy, initially associating high-confidence detection boxes followed by low-confidence ones, thereby effectively reducing worm mis-detection and false-positive rates while enhancing tracking continuity. Compared to StrongSORT, ByteTrack demonstrates superior performance in occlusion handling, data association, computational efficiency, and implementation simplicity.

Moreover, most research efforts typically conclude after successfully establishing deep learning-based worm detection and tracking systems, without further exploration of behavioral analysis. In some studies, only body bending angle measurements are performed and require extensive manual labeling. Or only computation of individual nematodes can be achieved and validation of complex behaviors such as reversed states is lacking [32–34]. However, this study presents an automated method for quantitative analysis of worm behavioral parameters, providing a comprehensive analytical platform for investigating behavioral dynamics. The proposed method enables automatic and rapid extraction of multiple behavioral parameters, including bending angle, roll frequency, and turning behavior, without manual intervention. Compared to traditional manual calculation methods, the automated method significantly improves the speed and efficiency of data processing and reduces the time and cost required for manual calculations. This innovative system serves as an important tool for understanding C. elegans behavioral dynamics and offers a replicable methodological framework for model organism research as a whole.

The main contributions of this paper can be summarized in the following three points:

(1) An improved worm detection and tracking framework integrating YOLOv8 with ByteTrack has been developed. This framework maintains optimized inference speed while improving detection accuracy, making it particularly suitable for real-time C. elegans detection and tracking applications. The system successfully achieves worm detection and tracking in single-target, multi-target, and specified-target scenarios.
(2) The integration of the Convolutional Block Attention Module (CBAM) mechanism enables the network to process image data more efficiently, simultaneously reducing computational resource consumption and enhancing model performance and accuracy. By implementing the Wise Intersection over Union (WIoU) loss function, the framework adaptively adjusts sample weights during training, thereby increasing the model’s focus on challenging detection cases.
(3) Based on the framework presented in this paper, a comprehensive analysis of locomotion parameters, including speed, trajectory, bending angle, and roll frequency, can be performed. We have developed an automated high-throughput method for C. elegans behavioral analysis, establishing a comprehensive analytical platform for investigating worm behavioral dynamics that enables fully automated extraction of multiple locomotor behavioral parameters.

## 2 Datasets and implementation details

### 2.1 Data acquisition and data enhancement

The dataset used in this study was compiled from publicly from public sources [16,23] to ensure reproducibility and benchmarking consistency. The C. elegans strains used in this study included the wild-type reference strain N2, along with the mutant strains FK101 and EG7985. All strains were cultured at 22°C on fresh nematode growth medium (NGM) agar plates. To incorporate developmental diversity, worms at various stages, including all larval phases (L1 through L4) and adulthood, were selected and subjected to brightfield imaging for morphological observation and analysis [23]. The final curated dataset consists of 1,759 images, which were randomly partitioned into a training set (1,408 images), a validation set (180 images), and an independent test set (171 images). In total, the dataset contains over 12,000 annotated worm instances encompassing all developmental stages.

To further validate the model’s performance under real laboratory conditions, a small set of self-collected datasets was included. These datasets were recorded under controlled experimental conditions using wild-type (N2) animals cultivated with standard methods. Worms at different developmental stages from L4 to young adulthood were selected. Animals were grown at 20 °C on 2% (w/v) NGM agar plates seeded with Escherichia coli OP50. For image acquisition, worms were transferred to a 2% (w/v) NGM agar plate with or without OP50 lawn to record crawling behavior. To record swimming behavior, we transferred worms in petri dish with thin layer of liquid NGM. Partial video sequences were acquired using an Olympus SZX7 stereo microscope, which was paired with DP20 camera. A custom-built microscope paired with a Mshiwi SUA134GC/M industrial camera. Images were captured at varying magnifications (2× to 4×), under uneven illumination, intentionally introducing variability to test the robustness of our method.

**Fig 1:**
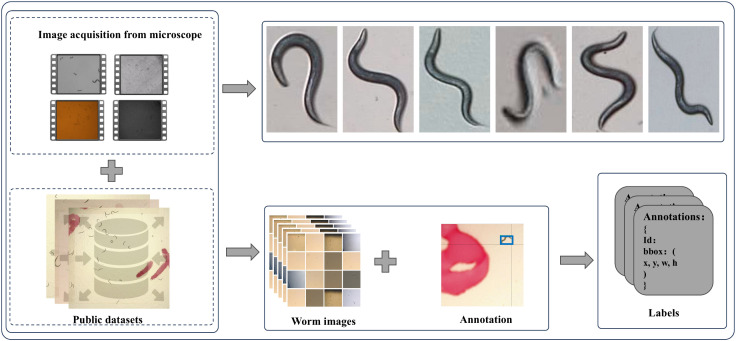
Workflow for image acquisition, data integration, and annotated dataset generation. The schematic illustrates the complete pipeline for constructing a labeled dataset for C. elegans behavioral analysis. (Top-left) Original worm images are acquired via microscopy. (Bottom-left) Existing public datasets, as symbolized by the database icon. (Top-right) Representative worm images are displayed. (Bottom-right) Annotation tools are used to label images, generating structured label files (e.g., YOLO-format TXT or JSON annotations). Arrows indicate the sequential flow of data processing and integration.

The datasets applied standard augmentation techniques, including rotation, translation, scaling, and cropping. Finally, all images were annotated using the Roboflow annotation platform. The complete workflow for dataset acquisition and construction is illustrated in the accompanying Fig 1.

### 2.2 Implementation details

The experimental setup utilized an NVIDIA GeForce RTX 3090 GPU with 24GB VRAM, CUDA version 11.7, and PyTorch framework implementation. The model was initialized with YOLOv8 pre-trained weights, configured with an initial learning rate of 0.05 and a batch size of 16. The optimization process employed Stochastic Gradient Descent (SGD) with momentum (0.9) and weight decay (1e-4). The training procedure consisted of 500 epochs. The reported results are based on 5 independent replicates.

## 3 Methodology

**Fig 2:**
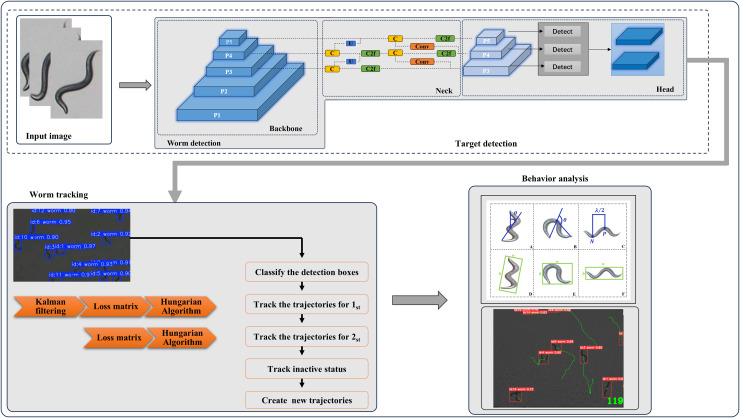
Framework for real-time detection and tracking of multiple worms. The framework consists of two main modules: an object detection network (top, blue) and a tracking and behavior analysis pipeline (bottom).

Accurate multi-target worm tracking in complex environments presents significant computational challenges. This study introduces an object detection and tracking framework that integrates modified YOLOv8 with ByteTrack, enabling real-time, precise worm tracking across diverse experimental conditions. The proposed framework architecture, illustrated in Fig 2, comprises two primary components: an object detection module and an object tracking module.

### 3.1 Worm detection based on the modified YOLOv8 Network

**Fig 3:**
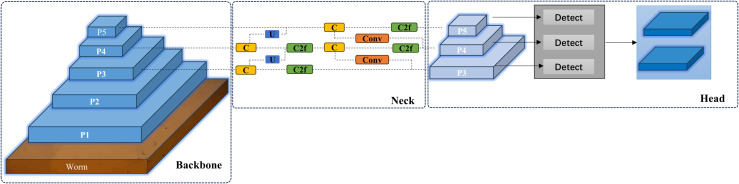
Worm Detection Module. The detection module processes input images through a multi-scale backbone and neck for feature extraction and fusion, generating detection results via the prediction head.

The worm detection module consists of three primary components: the backbone, neck, and head modules. The backbone module is responsible for extracting discriminative features from input images, while the neck module serves as an intermediate layer that integrates and enhances the features extracted by the backbone. The head module, functioning as the final layer, primarily performs bounding box regression for object detection. The worm detection module is shown in Fig 3 below.

**Fig 4:**
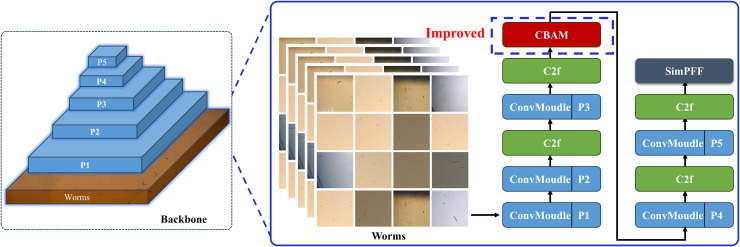
The backbone network of the worm detection module. Modifying the YOLOv8 detection architecture by integrating the Convolutional Block Attention Module (CBAM).

The backbone network serves as the fundamental component of the worm detection module, primarily responsible for extracting discriminative features from input image sequences and transforming raw images into comprehensive feature representations with rich semantic information. Shallow network layers capture basic visual characteristics including worm color, shape, and texture patterns, while deeper layers extract higher-level abstract features. These hierarchically extracted features form the essential basis for object detection in subsequent neck and head modules. The implemented backbone architecture follows the CSPDarknet [35] design paradigm. The backbone network architecture is illustrated in Fig 4, featuring a schematic overview of YOLOv8 on the left panel and a detailed layer-wise structural representation on the right panel. The numerical annotations adjacent to each module in the right diagram indicate the corresponding layer indices.

The ConvModule block comprises a convolutional layer (Conv), batch normalization layer (BN), and SiLU activation function, serving as the fundamental feature extraction unit. The C2f layer, a variant of the CSPNet [36] architecture, enhances computational efficiency through gradient redundancy reduction. This cross-module integration combines CSPNet with Bottleneck [37] structures, improving training efficiency via partial connections and feature fusion. The Bottleneck module consists of two convolutional layers and a residual network connection, implementing feature fusion through either concatenation or element-wise addition.

Spatial Pyramid Pooling Fast (SimPPF) comprises an initial convolutional layer, followed by multiple cascaded max-pooling layers, and a final convolutional layer that integrates multi-scale features. This architecture extracts multi-scale contextual information through hierarchical pooling operations, enabling the model to effectively capture worm morphological characteristics, including shape, size, and spatial location information, thereby enhancing target detection accuracy. To speed up the training, ReLU is used instead of SiLu in this work.

The neck network occupies the intermediate position between the backbone and Head networks, incorporating design principles from the PANet architecture. Through the implementation of a bottom-up pathway, it facilitates the propagation of shallow features, characterized by strong spatial localization but limited semantic content, to deeper network layers that exhibit robust semantic representation despite weaker spatial awareness. This architectural component enhances model performance by performing hierarchical feature fusion and refinement of the abstract features extracted from the backbone network, subsequently transmitting the optimized feature representations to the head network.

The head network serves as the decision-making component of the worm detection framework, responsible for generating final detection outputs that include both classification results and quantitative information.

#### 3.1.1 Adding attention mechanisms to improve YOLOv8.

**Fig 5:**
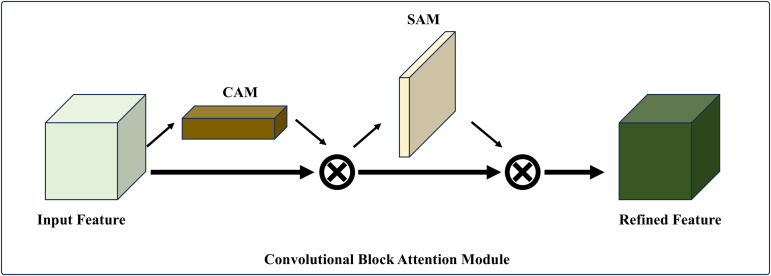
Convolutional block attention module.

In computer vision, attention mechanisms represent computational strategies that enable neural networks to selectively focus on salient local information while suppressing irrelevant features, thereby enhancing model performance. The implementation of attention mechanisms facilitates more efficient image data processing, optimizing computational resource utilization while simultaneously improving model accuracy and performance. Convolutional Block Attention Module [38] (CBAM) integrates two complementary attention components: the Channel Attention Module (CAM) and the Spatial Attention Module (SAM). In our implementation, CBAM adopts a sequential architecture where CAM precedes SAM, and this combined module is strategically integrated into the backbone network, as illustrated by the blue dashed box in Fig 5.

The channel attention mechanism operates by computing inter-channel relationships to differentiate feature importance across channels. Within the channel feature dimension, CBAM initially applies both average pooling and max pooling operations to the input feature map. These pooled features are subsequently processed through a shared fully connected layer, generating a channel attention weight vector. This vector is then utilized to perform channel-wise multiplication with the input feature map, effectively amplifying relevant channel features while attenuating less significant ones.

The spatial attention module computes pixel-wise attention weights by processing the input feature map. Initially, it employs global average pooling to extract feature vectors for each pixel position. These vectors are subsequently processed through a fully connected layer to generate spatial attention weights. The resulting weight matrix is then applied to perform element-wise multiplication with the input feature map, thereby emphasizing salient regions while suppressing less informative areas within the image.

#### 3.1.2 Modifying the loss function of YOLOV8.

The bounding box regression loss (Bbox_loss) quantifies the discrepancy between predicted and ground truth bounding box coordinates. This loss function computes the sum of squared differences between predicted and actual coordinate values, implementing an error-sensitive penalty mechanism that proportionally increases with prediction inaccuracy. Such design facilitates rapid correction of significant prediction errors during model training.

Intersection over Union (IoU) represents a widely adopted evaluation metric in object detection tasks, quantifying the spatial overlap between predicted and ground truth bounding boxes. However, the conventional IoU metric exclusively considers the overlapping region while disregarding other spatial relationships between the boxes, potentially introducing evaluation bias. This limitation becomes particularly problematic in worm detection scenarios, where frequent organism overlaps during movement significantly compromise the reliability of IoU-based assessments.

Given the complex movement patterns of worms, the constructed dataset inherently contains a substantial proportion of low-quality images, which may adversely affect the model’s generalization capability. Wise-IoU (WIoU) implements a dynamic non-monotonic focusing mechanism within its bounding box loss function, effectively addressing performance degradation caused by frequent worm overlaps during movement patterns. In this work, WIoU is used to replace the loss function inherent in YOLOv8.

### 3.2 Worm tracking based on ByteTrack

To enable comprehensive and precise behavioral analysis of worms, accurate tracking of complete skeletal structures and body contours is essential, rather than merely monitoring centroid positions. Current worm tracking methodologies offer a spectrum of capabilities. On one end, tools like Tierpsy provide comprehensive solutions capable of multi-organism tracking with varying levels of detail [14], from centroid-level to more elaborate features. On the other end, specialized trackers focus on high-fidelity skeletal and contour tracking, typically for single organisms. A persistent challenge in the field is developing a method that seamlessly integrates the scalability of multi-worm tracking with the detailed morphological and behavioral profiling offered by single-worm analyzers, particularly under demanding conditions such as frequent occlusion and overlap.

**Fig 6:**
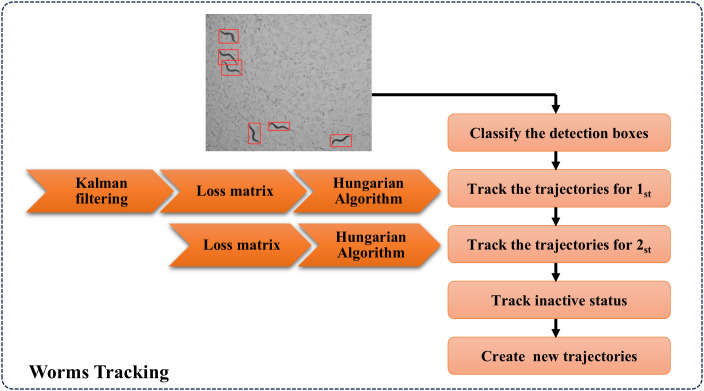
Worm Tracking Module. The tracking module associates detected targets across frames using Kalman filtering and the Hungarian algorithm, with additional logic to manage trajectory initiation and inactive states. Behavioral analysis is performed based on the resulting trajectories.

Conventional detection models frequently exhibit worm misidentification during frame loss events or prolonged occlusion scenarios in video sequences. ByteTrack effectively addresses these limitations. In this study, we implement the ByteTrack algorithm for worm tracking, with the complete tracking workflow illustrated in Fig 6.

ByteTrack represents a detection-based multi-object tracking algorithm, whose core innovation lies in its Byte data association strategy. Rather than discarding low-confidence detection results, the algorithm leverages spatial-temporal similarities between detection boxes and existing trajectories to distinguish foreground objects from background in low-score detections. This approach effectively minimizes detection omissions while enhancing motion trajectory consistency. In this study, ByteTrack utilizes bounding box outputs from the worm detection module for organism tracking. The algorithm implements distinct processing strategies for high-confidence and low-confidence detection boxes, effectively addressing worm occlusion challenges during movement while maintaining robust identity preservation and achieving efficient tracking performance. The steps of ByteTrack algorithm to track worms include:

(1) Trajectory and Bounding Box Classification: All tracking trajectories are categorized into active and inactive states, while bounding boxes in the current frame are classified based on confidence scores into high-score and low-score groups.
(2) Primary Matching: High-score bounding boxes are matched with active trajectories. The Kalman filter predicts the position and dimensions of bounding boxes for the subsequent frame, followed by IoU computation and optimal assignment using the Hungarian algorithm.
(3) Secondary Matching: Remaining active trajectories undergo matching with low-score bounding boxes, again employing the Hungarian algorithm for optimal assignment.
(4) Inactive Trajectory Handling: Newly emerged trajectories are matched with previously inactive trajectories.
(5) Trajectory Initialization: Unmatched high-score bounding boxes initiate new tracking trajectories.
(6) Result Output: The algorithm returns all successfully tracked trajectories.

**Fig 7:**
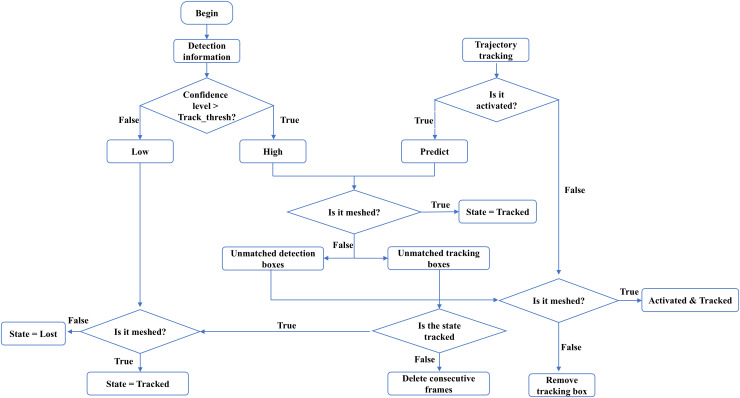
Worm tracking process diagram.

The algorithm flowchart is shown in Fig 7 below.

## 4 Results

In this study, we employed video sequence analysis for comprehensive worm detection and tracking, enabling detailed behavioral analysis of worm movement patterns. Prior to each recording session, the imaging plates were carefully positioned to ensure complete coverage of all worms within the camera’s field of view. To validate the efficacy of our proposed algorithm, we conducted comparative evaluations against several state-of-the-art models, including Deep-Worm-Tracker, and YOLOv7.

### 4.1 Evaluation indicators

This study selected mean Average Precision (mAP), Average Precision (AP), precision, recall, F1 score, and frames per second (FPS) as performance metrics for evaluating the deep learning model. The evaluation metrics were calculated using the following formulas.


$$Precision=\;\frac{TP}{TP+FN}\times \text{1}\text{0}\text{0}\%$$ (1)

Precision indicates the rate of accuracy, where TP (True Positives) represents the number of correctly identified worm images, FP (False Positives) denotes the number of non-worm images incorrectly detected as positive, and FN (False Negatives) indicates the number of missed worm images. Recall refers to the recall rate.


$$Recall=\frac{TP}{TP+FN}\times \text{1}\text{0}\text{0}\%$$ (2)

The F1 score is a means of striking a balance between precision and recall.


$$F_{\text{1}}=\frac{\text{2}\times Precision\times Recall}{Precision+Recall}\times \text{1}\text{0}\text{0}\%$$ (3)

Precision and recall values are utilized to construct the precision-recall curve (PR curve), with the area under this curve denoted as AP (Average Precision).


$$AP=\int_{\text{0}}^{\text{1}}P(R)dR$$ (4)

$$mAP=\frac{\sum_{i=\text{1}}^{n}{AP}_{i}}{n}$$ (5)

The mAP refers to the average AP. T denotes the detection time for a single image. FPS represents the number of images detected per second.


$$FPS=\frac{\text{1}}{T}$$ (6)

### 4.2 Results of worm detection and tracking

Experimental results demonstrate that Deep-Worm-Tracker, YOLOv7, and our proposed algorithm all exhibit robust performance in single-worm tracking scenarios. However, performance variations emerge among these algorithms when tracking multiple worms simultaneously. The proposed algorithm demonstrates superior performance in challenging scenarios involving frame loss or severe worm occlusion, with comparative results presented in Table 1.

**Table 1:** Comparison of different algorithms for worm tracking. All methods were executed on identical hardware (as detailed in Section 2.2) and evaluated on the same dataset using an image size of 1024 × 1024 pixels. The reported results are based on five independent replicates.

Model	Precision	Recall	F1 Score	mAP50	FPS
Deep-worm-tracker	0.987	0.972	0.979	0.981	87
YOLOV7	0.991	0.981	0.985	0.986	131
Ours	0.995	0.987	0.991	0.996	153

Table 2 evaluates the impact of integrating the CBAM attention module and the W-IoU loss function into a baseline YOLO model using a controlled variable approach (where “✓” denotes module inclusion). The experimental results demonstrate that employing CBAM alone improves the Recall to 0.986 and the F1 Score to 0.989, while using W-IoU alone significantly enhances mAP50 to 0.993. The combined use of CBAM and W-IoU yields the best overall performance, achieving a Precision of 0.995, Recall of 0.987, F1 Score of 0.991, and mAP50 of 0.996. These findings indicate that the two modules exhibit a synergistic effect in improving both detection accuracy and target recall capability.

**Table 2:** Ablation experiment results of the Modified-YOLO model.

Baseline	CBAM	W-IoU	Precision	Recall	F1 Score	mAP50	FPS
√			0.991	0.983	0.986	0.987	154
√	√		0.993	0.981	0.987	0.991	153
√		√	0.992	0.986	0.989	0.993	154
√	√	√	0.995	0.987	0.991	0.996	153

**Fig 8:**
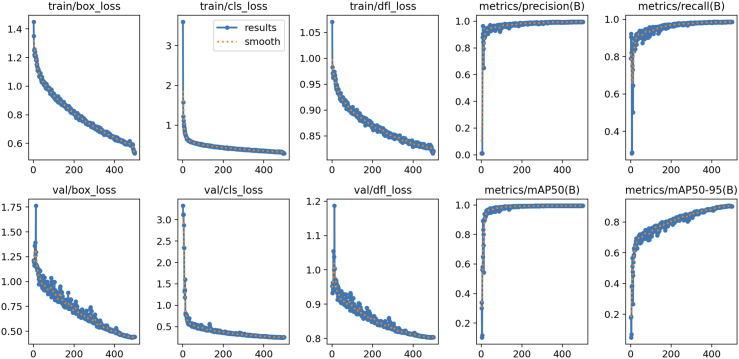
Training result graph of proposed framework.

Comparative analysis reveals that the proposed model achieves superior performance metrics, including accuracy, recall, F1 score, and mAP, outperforming existing object detection and tracking models. These results demonstrate the model’s enhanced robustness and precision in minimizing false positive errors and maximizing correct classification rates. Furthermore, the FPS measurements indicate significantly improved detection speed compared to other models. Fig 8 illustrates the training dynamics of the proposed model. As training progresses, the bounding box loss (box_loss), classification loss (cls_loss), and distribution focal loss (dfl_loss) exhibit consistent convergence behavior. Both mAP50 and mAP95 metrics demonstrate rapid improvement during initial training cycles, eventually reaching stable performance levels as training continues.

**Fig 9:**
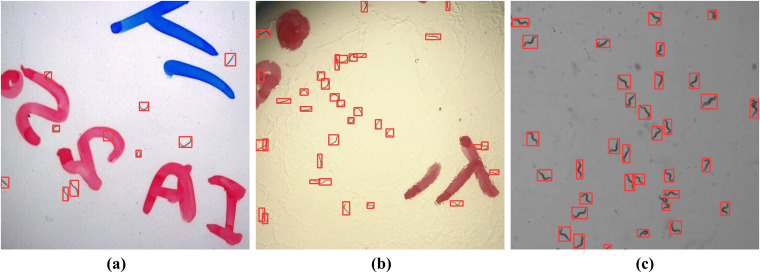
Worm detection under different backgrounds.

The proposed worm tracking model demonstrates high training efficiency, requiring 15% less training time, while achieving accurate real-time performance in both online and offline scenarios. It maintains tracking accuracy under challenging conditions, including occlusion and overlap among worms. As shown in Fig 9, the model delivers reliable detection when nematodes are spatially separated and remains effective under partial body overlap where individuals remain distinguishable. However, performance declines in cases of extensive overlap where worms are nearly fused into a single entity. These results indicate that the algorithm is robust to background interference and variably oriented partial overlaps, yet struggles with severely fused nematode pairs exhibiting high degrees of overlap.

**Fig 10:**
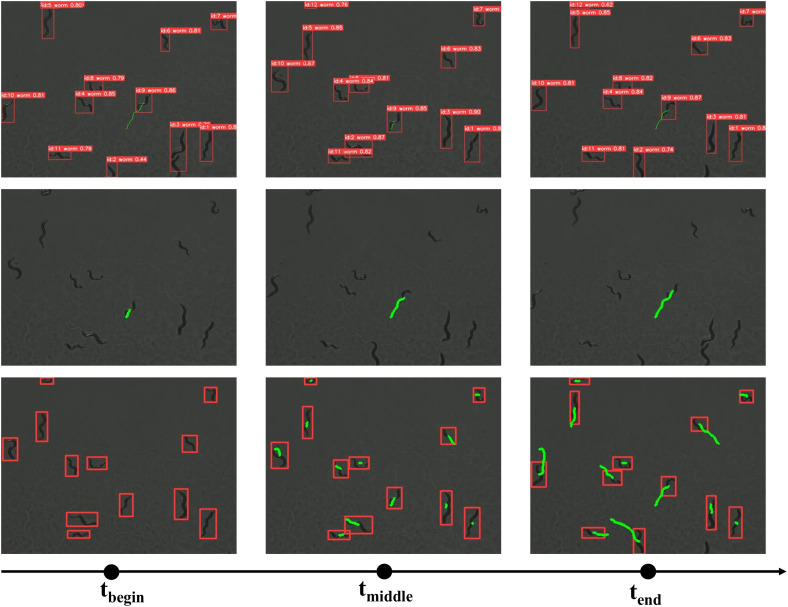
Multiple worms tracking trails.

The system provides programmable output configurations for customizable visualization, allowing users to selectively display tracking trajectories—such as those of a single worm (specified by ID), multiple designated worms, or the entire population—while the underlying detection and tracking algorithm processes all worms in the image simultaneously. As demonstrated in Fig 10 the proposed framework facilitates real-time trajectory visualization based on complete skeletal tracking of multiple worms simultaneously.

### 4.3 Basic motility parameters of worms

Locomotion speed, forward movement, and backward movement constitute fundamental behavioral parameters in C. elegans. These metrics serve dual purposes: distinguishing between active and stationary states, and identifying sudden accelerations (movement bursts). The proposed framework supports computation of these basic locomotion parameters for both single-worm and multi-worm scenarios.

The object detection results provide comprehensive spatial information, enabling precise calculation of worm movement speed through coordinate analysis. It is important to note that the speed calculation focuses solely on magnitude, disregarding directional components. For discretely sampled video data, instantaneous speed is calculated as the displacement between consecutive frames divided by the inter-frame interval, while average speed is computed over multiple frames using the total displacement divided by the corresponding time interval. The displacement of worms between consecutive image frames is calculated as the spatial difference Δs, while the corresponding time interval between frames serves as Δt for instantaneous speed computation. Where $(x_{t},\;y_{t}$.


$$v=\frac{\Delta s}{\Delta t}=\frac{\sqrt{{\left(x_{t}-x_{t-\text{1}}\right)}^{\text{2}}+{\left(y_{t}-y_{t-\text{1}}\right)}^{\text{2}}}}{\Delta t}$$ (7)

Without considering direction, the average speed of worm movement can be obtained from equation 8.


$$\overset{―}{v}=\frac{\sum_{i=\text{1}}^{n}\Delta s_{i}}{\sum_{i=\text{1}}^{n}\Delta t_{i}}$$ (8)

The proposed framework facilitates precise calculation of individual worm movement speeds within the field of view. Each qualifying worm was represented with one value for average speed determined by the arithmetic mean of the values calculated each second.

The direction of worm movement (forward or backward) is determined based on the displacement vector relative to the worm’s intrinsic body orientation, defined by the head-to-tail axis. Forward movement occurs when the displacement is aligned with the head-to-tail direction, backward movement occurs when it is opposed..


$$Direction=\left\{ \begin{array}{c} Forward,\;tail\_to\_head\;\\ Backward,\;head\_to\_tail\; \end{array}\right.\;$$ (9)

We selected four C. elegans individuals as representatives to demonstrate their movement trajectories, locomotion speeds, and movement states (forward/backward movement). For each specimen, the first panel displays the worm’s trajectory path, the second panel presents its speed profile, and the third panel illustrates the movement state transitions.

**Trajectory and Behavioral Analysis**: The trajectory diagram (first panel) illustrates the worm’s positional coordinates (x, y) within the experimental arena. Continuous linear movement is represented by straight paths with uniform coloration, whereas curved or spiral trajectories featuring abrupt color transitions indicate sharp turning events. A color gradient scale (right) denotes instantaneous speed, with dark purple corresponding to low speed and yellow representing high speed - the latter suggesting potential escape responses or stimulus-evoked behaviors. This visualization enables quantitative assessment of locomotion patterns and behavioral states.

**Fig 11:**
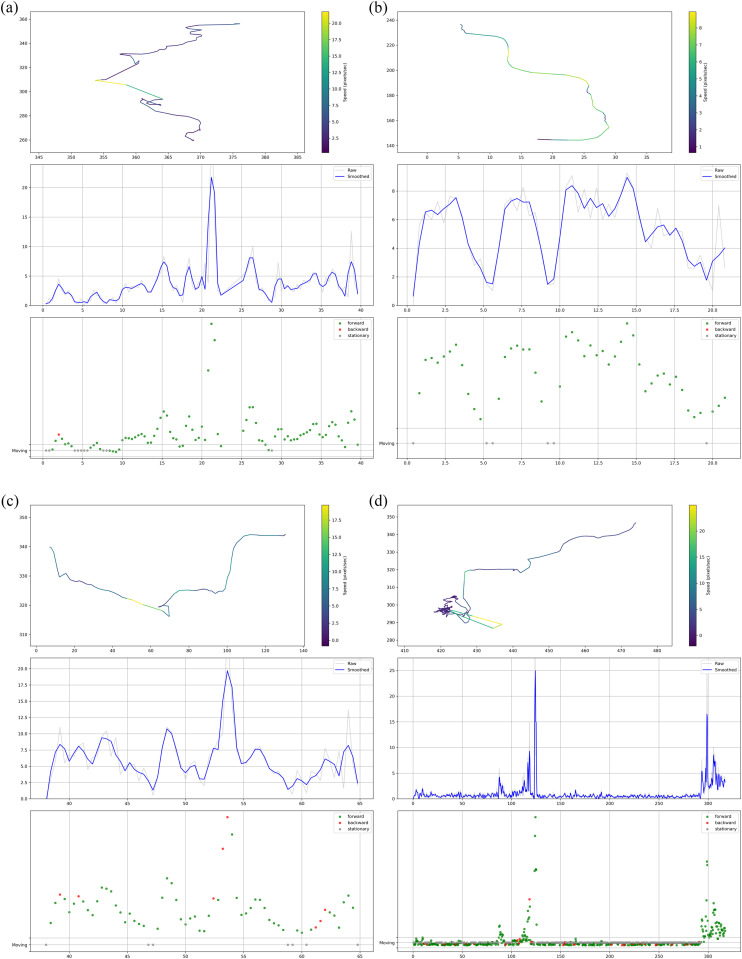
Movement trajectories, speeds, and movement states of C. elegans.

**Speed Profile Characterization**: The speed time series (second panel) presents the worm’s speed (pixels/second) over time (seconds), displaying both raw data (grey line, containing noise) and filtered data (blue line, showing the underlying trend). Persistent speed minima correlate with turning events or quiescent phases (Fig 11d), while prolonged troughs combined with visual tracking confirm abnormal states such as mortality. Speed peaks reflect sprinting episodes, with excessive signal jaggedness indicating the need for smoothing window parameter optimization to better resolve the true kinematic profile.

**Integrated Motion Analysis**: When time-aligned across panels, the locomotion state diagram (third panel) reveals coordinated behavioral transitions: green markers indicate forward locomotion, red markers backward locomotion, and grey markers stationary periods. Cross-referencing speed extrema with corresponding trajectory positions shows consistent spatiotemporal relationships - speed reversals and trajectory inflections precisely coincide with abrupt locomotion state transitions, demonstrating the system’s capability to capture complete kinematic profiles and behavioral sequences.

The movement trajectories, speeds, and behavioral states of the nematodes were further visualized as shown in Fig 11. It is important to note that the four trajectories presented are a representative subset. The algorithm developed in this work is capable of simultaneously tracking and visualizing a large number of individuals. However, to facilitate a clear interpretation of individual movement patterns and to avoid visual clutter arising from overlapping trajectories, the present study selectively displays representative examples. This selective visualization strategy ensures that qualitative and quantitative information about movement states and spatial preferences remains clearly accessible.

### 4.4 Advanced locomotor behavioral parameters of worms

As a widely used and studied model organism, C. elegans worms offer the ability to investigate implications of behavioral change. Most research efforts typically conclude their investigations upon achieving successful implementation of deep learning-based worm detection and tracking systems, without further exploration of behavioral analysis. In contrast to conventional approaches, this study provides an extended quantitative analysis of advanced locomotor behaviors in worms, including rolling frequency, turning behavior, and undulation frequency. These motion parameters not only possess individual biological significance but also enable a more comprehensive behavioral pattern analysis when combined.

#### 4.4.1 Automated behavioral parameter extraction.

**Fig 12:**
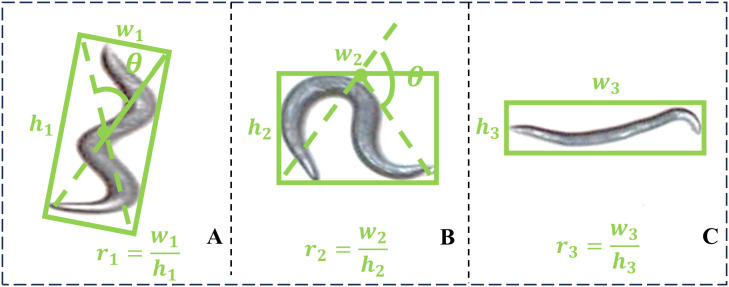
Bending angle calculation. A–C shows representative examples corresponding to three bending morphology categories. While these examples illustrate the most commonly observed morphologies, they do not encompass the full spectrum of possible body shapes.


For the bending angle of worms, this study introduces an automated high-throughput method that estimates worm bending angles using detected bounding boxes. The proposed framework supports both single-worm and multi-worm tracking modes, facilitating efficient extraction of bounding box dimensions (width and height). Furthermore, the system enables morphology-specific bending angle calculation methods based on real-time worm tracking visualizations. The bending angle estimation strategy adapts to worm morphology:

(1) For morphology depicted in Fig 12A, the diagonal angle of the bounding box is computed.

$$\theta =\text{2}\times arctan\frac{w_{\text{1}}}{h_{\text{1}}}$$ (10)

(2) For morphology shown in Fig 12B, the angle is calculated relative to the bounding box centroid.

$$\theta =\text{2}\times arctan\frac{{\text{2}\times h}_{\text{2}}}{w_{\text{2}}}$$ (11)

(3) No angle calculation is performed for essentially straight worms as illustrated in Fig 12C.

Roll frequency parameter was calculated based on smoothed speed provided by WormLab [32]. This study implements an automated high-throughput approach for quantifying worm roll frequency. Leveraging the proposed tracking framework, the width (w) and height (h) of worm bounding boxes are readily obtained. We define the aspect ratio r = w/h and monitor its temporal variation. An important assumption underlying curvature calculation is that the bounding box tightly encloses the nematode’s body. This requirement is met by the YOLO architecture, whose inherent design advantages—including anchor box matching, precise coordinate regression, and a loss function that jointly optimizes overlap, center distance, and aspect ratio—contribute to accurate detection. Coupled with Non-Maximum Suppression, these features enable YOLO to generate bounding boxes that fit closely around the nematode. A roll event is identified when the aspect ratio meets the criterion defined in equation 11, and the corresponding roll frequency can be subsequently calculated using equation 12.


$$\begin{array}{c} Roll\;Event=\left\{ \begin{array}{c} @l\text{1},\;\left|\frac{w_{t}}{h_{t}}-\frac{w_{t-\text{1}}}{h_{t-\text{1}}}\right|>\theta_{roll}\\ \text{0},\;otherwise \end{array}\right.\; \end{array}$$ (12)

where $\theta_{roll}$ represents the threshold.


$$Frequency=\frac{\sum Roll\;Events}{T_{total}}$$ (13)

where $w_{t},\;h_{t}$.

**Fig 13:**
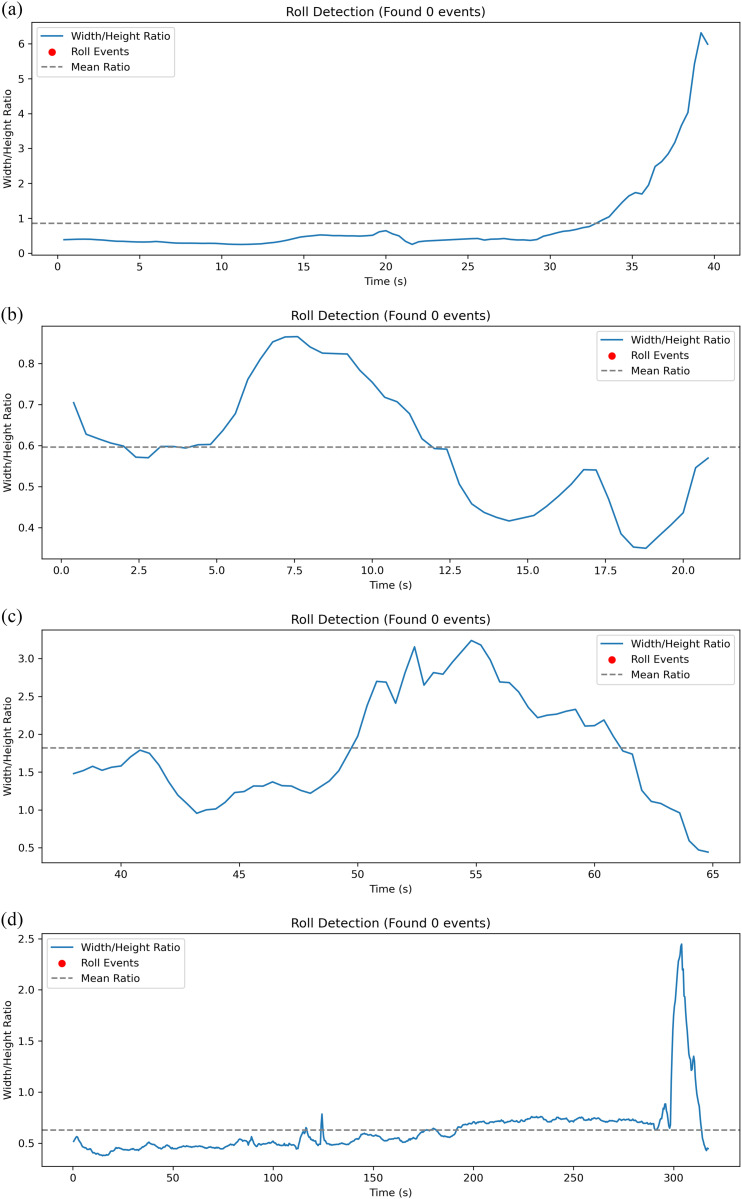
Roll event detection.

As illustrated in Fig 13, the horizontal axis represents time (in seconds), while the vertical axis denotes the aspect ratio (w/h) of the detection bounding box. The blue curve depicts the temporal variation of the aspect ratio, with red scatter points indicating detected roll events. For reference, a gray dashed line marks the mean aspect ratio value.

The Omega turn (Ω-turn) is a highly specific turning behavior in the worm C. elegans named after its movement trajectory resembling the Greek letter “Ω” (Omega). As a characteristic movement pattern of C. elegans, the Omega turn provides a simple yet powerful model for understanding neural regulation, behavioral decision-making, and disease mechanisms, holding broad value in both fundamental research and applied science.

Omega turns are obtained by calculating curvature integrals:


$$S(t)=\int_{t-\Delta \text{t}}^{t}\left\lceil k(\tau )\right\rceil d\tau$$ (14)

$$k(\tau )=\frac{{\dot{x}}_{t}{\ddot{y}}_{t}-{\dot{y}}_{t}{\ddot{x}}_{t}}{{\left({\dot{x}}_{t}^{\text{2}}+{\dot{y}}_{t}^{\text{2}}\right)}^{\frac{\text{3}}{\text{2}}}}$$ (15)

where, $\dot{x}$ and ${\ddot{x}}_{t}$ represent the first- and second-order derivatives of position, respectively, computed using the central difference method, a turn is identified when $k(\tau )>\theta_{curve}$

**Fig 14:**
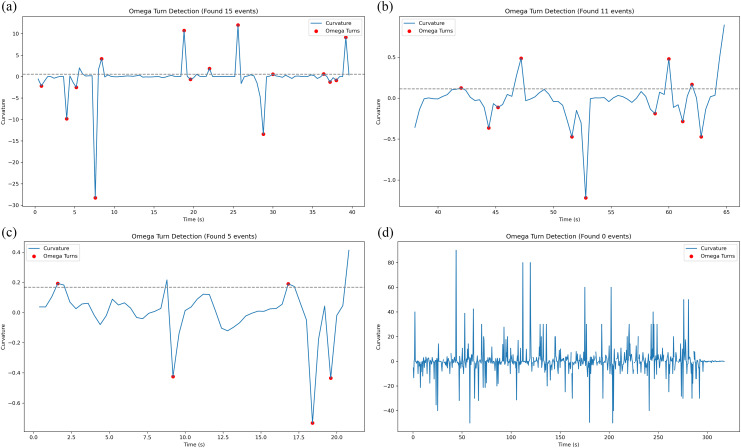
Omega turn detection of Worms.

Omega turn detection of worms as shown in Fig 14, where the horizontal axis represents time (in seconds) and the vertical axis indicates curvature. Positive curvature values correspond to leftward body bending, while negative values indicate rightward bending, with the absolute magnitude reflecting turning sharpness. The blue trace shows the raw curvature profile, representing instantaneous body bending intensity, while the gray dashed line denotes the curvature threshold. Red markers identify detected omega-turn events, where single-peak signatures characterize rapid omega turns and multi-peak patterns indicate sequential turning maneuvers.

#### 4.4.2 Composite analysis of multiple advanced locomotor behaviors.

The combined analysis of locomotor parameters (such as roll frequency and turning behavior) enables a more comprehensive understanding of C. elegans’ complex behavioral patterns, neural regulatory mechanisms, and pathological states through the integration of multidimensional movement characteristics.

**Fig 15:**
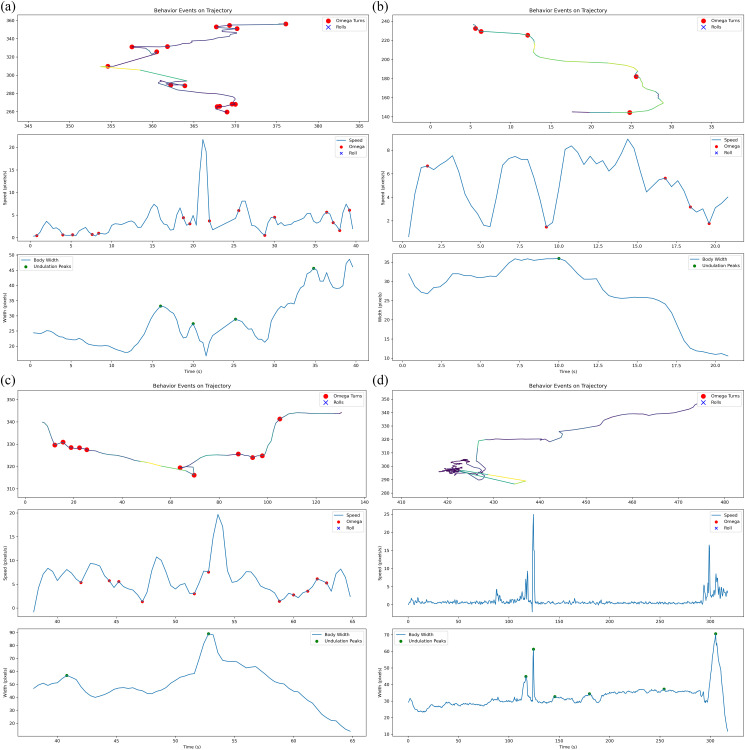
Analysis of multiple advanced locomotor behaviors.

Fig 15 provides an integrated visualization of the worm’s movement trajectory overlaid with key behavioral events (Omega turns, rolls, and undulation peaks), where the x- and y-axes represent spatial coordinates and the trajectory line (gray) shows the path. Colored markers highlight specific events: red circles for Omega turns (high-curvature reversals), blue crosses for rolls (body-width-ratio spikes), and green circles for undulation peaks (body-width maxima). The synchronized speed curve (bottom subplot) maps temporal dynamics, with event timestamps marked for correlation. This plot reveals spatial preferences, temporal patterns, and behavioral sequences, enabling rapid assessment of environment interactions or neural defects. For quantitative analysis, measure event densities in specific zones or compare timing relative to stimuli.

**Fig 16:**
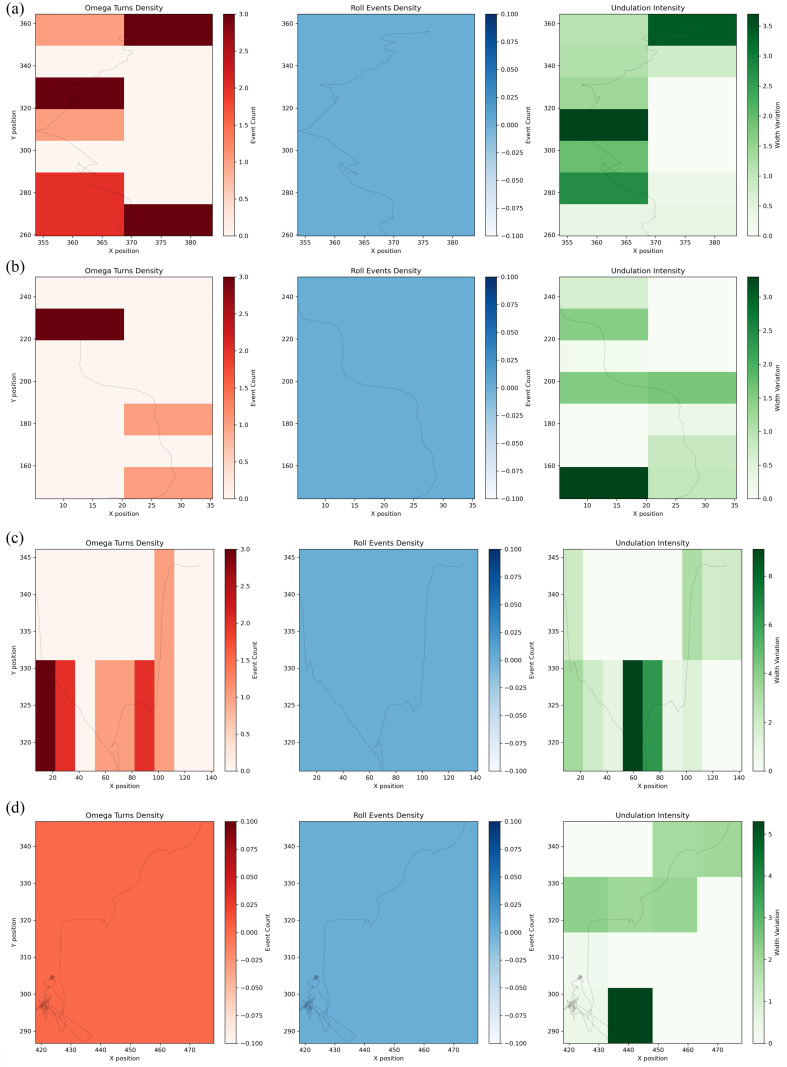
Heatmaps for combined analysis of worm motion parameters.

The behavioral heatmap, as shown in Fig 16, (left/middle/right) visualizes spatial densities of distinct locomotion patterns: the left heatmap (Reds) shows Omega turn hotspots, highlighting regions where rapid reversals cluster; the middle heatmap (Blues) maps rolling event density, indicating areas where body flips occur; and the right heatmap (Greens) displays undulation intensity, reflecting muscle activity strength across the arena. To analyze, identify overlapping hotspotsor track gradient patterns (e.g., directional shifts in undulation suggesting chemotaxis). Use color intensity to quantify event frequency (darker = more frequent) and compare across conditions. Always cross-reference with trajectory data to contextualize localized behaviors.

**Fig 17:**
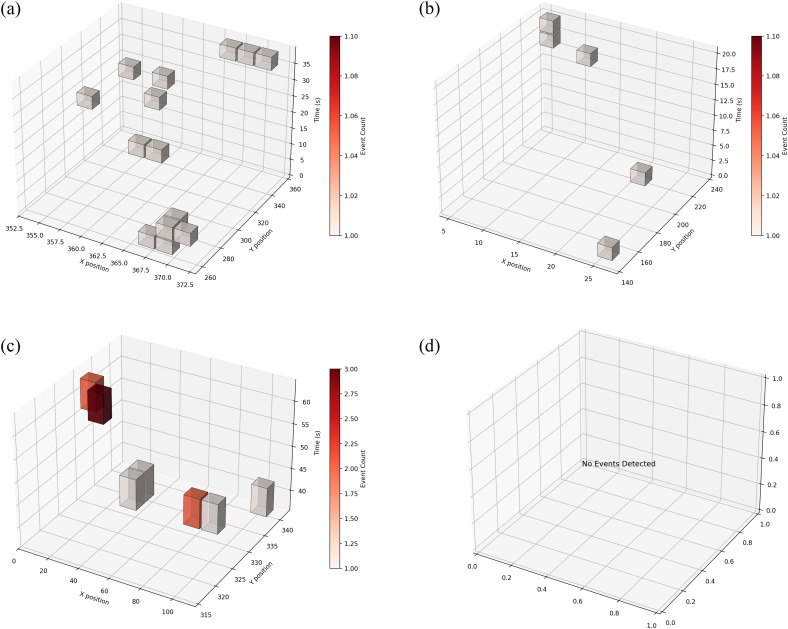
Three-dimensional thermograms for combinatorial analysis of worm motion parameters. (a)–(c) 3D density distributions of different behavior categories (e.g., reversal turns, rolling, undulation), with color scale indicating high-to-low event frequency across X-position, Y-position, and time. (d) Absence of events in cases of worm inactivity or abnormal states.

To analyze the left 3D heatmap, as shown in Fig 17, focus on identifying spatiotemporal clusters where dense red cubes indicate frequent Omega turns. Examine X-Y planes to locate spatial hotspots and Z-axis progression to detect timing patterns. Measure cube height/density to quantify turn frequency in specific zones, and compare slices at key timepoints to assess behavioral dynamics. High-density streaks along Z reveal persistent avoidance areas, while isolated cubes may indicate acute responses. Cross-reference with trajectory plots to confirm turn contexts. As shown in Fig 17(d), motion events cannot be detected for worms in abnormal states (stationary).

The roll frequency and turning behavior of C. elegans serve as microscopic yet quantifiable behavioral metrics, providing a unique window for interdisciplinary research. This multi-parameter synergistic approach not only distinguishes behavioral strategies under different environmental stimuli and reveals the integrative principles of sensorimotor neural circuits, but also demonstrates its core value in overcoming the limitations of single-parameter analysis. By quantifying dynamic correlations between parameters, it enables holistic understanding of biological motion decision-making systems, holding significant importance for both fundamental neuroscience and translational applications.

## 5 Discussion

The present study introduces an advanced worm tracking framework that integrates YOLOv8 with ByteTrack, achieving superior detection and tracking performance through optimized network architecture and state-of-the-art training strategies. This framework addresses the growing demand for high-throughput, automated behavioral analysis in C. elegans research, enabling precise quantification of complex locomotion patterns at scale.

A key innovation of our method is its ability to automatically extract multiple behavioral parameters, including movement speed, bending angle, and roll frequency, without manual intervention. Unlike traditional methods that rely on labor-intensive measurements, our framework supports simultaneous tracking of multiple worms while maintaining automated extraction of various behavioral parameters with high precision. The temporal consistency demonstrated in trajectory plots, speed profiles, and locomotion state diagrams (Figs 11 and 15) establishes a comprehensive analytical platform for investigating worm behavioral dynamics. While this study has demonstrated the robustness of our tracking framework on wild-type and several mutant strains from a public dataset, we acknowledge that its full potential for genetic screening can be further solidified by applying it to a broader spectrum of mutants with well-characterized locomotor phenotypes.

The research aspect ratio-based roll detection (Eqs. 12–13) offers a computationally efficient alternative to traditional skeleton analysis methods [3], albeit with a marginal trade-off in precision. Notably, curvature analysis revealed distinct kinematic signatures between single omega turns (sharp, single-peak events) and sequential turns (multi-peak patterns) (Fig 14), providing new insights into navigational decision-making. For the visual analysis of nematode behavior, the use of physical scales (e.g., micrometers or millimeters) improves interpretability, whereas pixel-based units facilitate precise spatial localization. It is therefore essential to adopt the appropriate unit system based on the specific requirements of the experimental or analytical context.

While this framework represents a significant advancement, several limitations remain: Tracking accuracy decreases at higher worm densities due to morphological overlaps exceeding detection capabilities. Future work will focus on (1) implementing closed-loop stimulation experiments in C. elegans and (2) developing systematic population-level quantification tools for group behavioral dynamics to enable more robust statistical comparisons and enhance ecological relevance. By leveraging the real-time tracking capability and automated behavioral analysis methods developed in this study, we aim to dynamically adjust stimuli based on the worms’ real-time behavioral feedback, thereby improving experimental efficiency.

Despite these constraints, the algorithmic advancements open new avenues for quantitative neuroethology, offering a robust platform for high-throughput behavioral phenotyping and real-time interactive experiments.

## 6 Conclusion

This study introduces a modified worm tracking framework that integrates YOLOv8 with ByteTrack, achieving improved detection and tracking accuracy through optimized network architecture and advanced training strategies. By implementing low-confidence detection box retention and trajectory-detection similarity matching, the framework effectively addresses challenges associated with temporary C. elegans worms disappearance and occlusion events, significantly reducing both false negatives and false positives while enhancing tracking continuity and robustness. Comparative evaluation demonstrates that the proposed worm detection framework outperforms existing models across multiple performance metrics.

Furthermore, this study advances the standardization of C. elegans behavioral parameter analysis through detailed investigation of worm locomotion patterns. The automated high-throughput behavioral analysis method can eliminate the subjectivity of manual observation and ensure the quantitative consistency of behavioral parameters (e.g., bending angle, roll frequency, etc.). Notably, the system enables simultaneous analysis of multiple worms’ behavioral data, significantly enhancing experimental throughput to accelerate high-throughput drug screening or gene function studies. Future research should focus on the development of high-precision, fully automated and high-throughput solutions for dense C. elegans populations.
